# Antibacterial and Photocatalytic Coatings Based on Cu-Doped ZnO Nanoparticles into Microcellulose Matrix

**DOI:** 10.3390/ma15217656

**Published:** 2022-10-31

**Authors:** Mariana Bușilă, Viorica Mușat, Rodica Dinică, Dana Tutunaru, Aida Pantazi, Dorel Dorobantu, Daniela C. Culiță, Marius Enăchescu

**Affiliations:** 1 LNC-CNMF—Center of Nanostructures and Functional Materials, Faculty of Engineering, “Dunărea de Jos” University of Galati, 111 Domneasca Street, 800201 Galați, Romania; 2 Department of Chemistry, Physics and Environment, Faculty of Sciences and Environment, “Dunărea de Jos” University of Galati, 800201 Galați, Romania; 3 Faculty of Medicine and Pharmacy, “Dunărea de Jos” University of Galati, Street, 800, 800201 Galați, Romania; 4 CSSNT—Center for Surface Science and Nanotechnology, University Politehnica of Bucharest, 313 Splaiul Independentei, 060042 Bucharest, Romania; 5 S.C. NanoPRO START MC S.R.L., Mitropolit Antim Ivireanu Street 40, 110310 Pitesti, Romania; 6 Institute of Physical Chemistry “Ilie Murgulescu” of Romanian Academy, 060021 Bucharest, Romania; 7 Academy of Romanian Scientists, Splaiul Independentei 54, 050094 Bucharest, Romania

**Keywords:** Cu-doped ZnO, microcellulose, polyester/cotton fiber, nanostructured hybrid coating, photocatalytic activity, antimicrobial activity

## Abstract

The paper presents a successful, simple method for the preparation and deposition of new hybrid Cu-doped ZnO/microcellulose coatings on textile fibers, directly from cellulose aqueous solution. The morphological, compositional, and structural properties of the obtained materials were investigated using different characterization methods, such as SEM-EDX, XRD, Raman and FTIR, as well as BET surface area measurements. The successful doping of ZnO NPs with Cu was confirmed by the EDX and Raman analysis. As a result of Cu doping, the hybrid NPs experienced a phase change from ZnO to (Zn_0.9_Cu_0.1_)O, as shown by the XRD results. All the hybrid NPs exhibited a high degree of crystallinity, as revealed by the very sharp reflections in XRD patterns and suggested also by the Raman results. The evaluation of the very low copper-doping (0.1–1 at.%) effect has shown different behavior trends of the hybrid coatings compared with the starting oxide NPs, for MB and MO photodegradation. Continuous increases up to 92% and 60% for MB and MO degradation, respectively, were obtained at maximum 1 at.%-Cu doping coatings. Strong antibacterial activity against *S. aureus* and *E. coli* were observed.

## 1. Introduction

There is an increasingly major concern about bacterial contamination in the public health field. In this regard, substantial efforts have been focused to obtain highly efficient nanocomposite coatings with antimicrobial and photocatalytic properties that could effectively prevent the growth and spread of bacterial infection [1,2]. The use of cellulose as a matrix to obtain semiconductor metal oxide-based composites has been an efficient approach to increase the functionality, durability, biodegradability, and recyclability of textiles. Due to their good mechanical properties (e.g., high tensile strength) at ambient temperature, biodegradability, biocompatibility, chemical stability and high crystallinity, cellulose derivatives have many advantages in this respect [2,3,4,5,6]. The association in various ways of semiconductor metal oxide nanoparticles (MONPs) with cellulose has been intensively studied for applications in many fields. Using a large number of inorganic and organic fillers such as nanoparticles (NPs), nanowires (NWs), nanofibers or nanosheets, micro/nanocellulose-based materials were investigated for applications such as water remediation [7], flexible electronics or smart packaging and functional clothing [8]. In the last years, an accelerated development was observed in biomedical applications (biosensors, artificial skins, bioelectronics, and drug delivery [9,10]. Nanocrystal cellulose (NCC), nanofibril cellulose (NFC) and bacterial cellulose (BC) are among the most intensively used nanobiomaterials, both as the matrix and reinforcing element. The development of nanostructured hybrid composites, including biocompatible and bioactive metal oxides nanomaterials and cellulose-based derivatives with biomedical functionalities are of high interest in the biomedical field [2,11,12,13].

The nanometric size and the high specific surface area of nanoparticles are directly correlated with the efficiency of their photocatalytic and antimicrobial properties [8]. As known, the agglomeration into larger particles due to the inter-particle dipole forces represents the main drawback of the efficient use of MONPs. Their dispersion as particles with dimensions below 50 nm by ex-situ processes or in-situ synthesized quantum dots (≤20 nm), can be efficiently obtained through heterogeneous nucleation on the active surfaces of solid materials. The dispersion of MONPs on different cellulose nanostructures, which is among the most versatile and sustainable natural resources, has led to new strategies and perspectives for its use in new emerging areas [8]. Based on an association of beneficial functional properties coupled with low cost and toxicity, ZnO nanostructures were incorporated into many biocompatible products, such as cosmetics, sunscreens as UV-blockers, photo-catalysis, medicines, hospital fabrics and more [13,14,15,16]. ZnO quantum dots (below 10 nm) demonstrated powerful properties against a wide range of bacterial organisms [17] and have promoted biomedical research for the treatment of cancer [14,15,16,18]. Recently, cellulose-based fabrics covered with ZnO nanoparticles having different morphologies (0D, 1D-rods and 3D-stars) showed selective antimicrobial and photocatalytic activity. Thus, the cotton fabric treated with 0D particles (26 nm) showed high antimicrobial efficiency and those with 1D nanostructures exhibited the highest UV protection [19]. The control of semiconductor MONPs’ size and shape can be achieved by using surfactants or doping with metallic elements, such as Cu, Mn, Fe, etc., which allow tuning their band gap energy and, subsequently, controlling and enhancing their functional efficiency [7,20,21,22,23]. Composites based on micro/nanocellulose containing ZnO and CuO, but also TiO_2_, MgO or Fe_2_O_3_ were synthesized and studied to replace antibiotics as antimicrobial agents against resistive bacteria [8]. ZnO/Cu bi-layered coatings on the cotton fabrics were investigated and have proven effective self-cleaning and antimicrobial properties in novel Corona discharge treatment [8,24]. Durable, multifunctional tricomponent AgNPs/ZnONPs/CuNPs coatings for cotton fiber functionalized with polyethyleneimine have shown very good photocatalytic and antimicrobial activity together with high electrical conductivity [25]. As mentioned before, controlled syntheses are required to obtain the targeted nanometric size, shape and crystalline pattern of nanocatalysts, which correlate the obtained properties with the targeted functions [13,26,27]. Physical, chemical and, more recently developed, biological methods were reported for the synthesis of undoped and doped MONPs and related composites, including textile coatings [11,28,29,30]. Chemical solution-based routes such as controlled precipitation, sol-gel and solvo/hydrothermal methods are among the most widely used for these materials [1,11,12,31,32,33]. Modified textile materials with colloidal solutions containing inorganic MONPs, for domestic and industrial use, with environmentally friendly functionalities, such as self-cleaning, antibacterial, and photocatalytic properties, have developed rapidly in recent years [3,16,34,35,36,37]. This is a successful sol-gel approach, which uses the so-called “nanosols” as innovative coating agents for the functionalization and treatment of textile materials, that can be deposed by various methods such as padding, dipping, or spraying [38]. The resulted sol-gel coatings consist of a three-dimensional network of the self-assembled nanoparticles remaining after solvent evaporation [37]. By the nanosols-based technology, innovative biofunctional textiles with applications in the fields of biosensors, bioreactors, or drug release systems, as well as photocatalytic and light-responsive materials were obtained [12,38]. Some authors also reported the use, during solution-based synthesis, of the cross-linking agents to increase the interactions between MONPs and cellulose fibers, and subsequently to increase coating’s efficiency and durability and to protect textile fibers against UV radiation [39]. However, there are relatively few reports on undoped and doped-ZnO nanoparticles in cellulose matrix [28,29,30,31,32,33,34,35,36,37,38], and only a few reports regarding the preparation of ZnO-based/MC hybrids directly from aqueous or mixed water-alcohol solutions [37,39].

To the best of our knowledge, only a few published works report the preparation of cellulose/ZnO-based nanocomposites directly from cellulose aqueous solution, as it represents a major challenge due to the numerous -OH groups present on the cellulosic chains, which cause strong intra and intermolecular interactions through hydrogen bonds [10]. This paper presents the preparation and characterization of new Cu:ZnO/MC hybrid coatings on textile fibers (polyester/cotton–50%/50%) deposed by a modified sol-gel route assisted by GPTMS organosilane crosslinking agent, directly from an aqueous solution of microcellulose. The Cu:ZnO NPs, prepared by hydrolysis in the aqueous solution of zinc nitrate and copper nitrate in the presence of sodium hydroxide (NaOH), were dispersed into an aqueous solution of microcrystalline cellulose (MC). After GPTMS addition, the colloidal dispersions were applied to the textile fibers by immersion. The obtained coatings were stabilized in the air at moderate temperature (120 °C) and investigated morphologically, compositionally, and structurally investigated by SEM-EDX, XRD, Raman and FTIR techniques and tested for antibacterial and photocatalytic properties. Direct use of a silane (GPTMS) in the aqueous MC solution containing ZnO-based nanoparticles with very low Cu-doping (0.1–1 at.%) to ensure very good dispersibility of the latter in solution but also for very good adhesion of the hybrid coatings to the textiles, represents the novelty of this study.

## 2. Materials and Methods

### 2.1. Materials

Zinc nitrate hexahydrate Zn(NO_3_)_2_∙6H_2_O (≥98%), copper (II) nitrate trihydrate Cu(NO_3_)_2_∙3H_2_O (≥99%) and 3-glycidyloxypropyltrimethoxysilane (GPTMS) were purchased from Sigma-Aldrich (Darmstadt, Germany). The microcrystalline cellulose 102 NF (Fine) used as a matrix was provided by Chemical Store. Potassium hydroxide (reagent grade 90%) was supplied by Merck (Darmstadt, Germany) and the ethanol (99.3%), used as a solvent for the preparation of the solutions, was purchased from Chemical Company (Iasi, Romania). The broth and agar nutrients, as well as the blank discs used for investigating the antimicrobial activity, were purchased from Merck (Darmstadt, Germany) and Bioanalyse (Ankara, Turkey), respectively. Deionized water was used as a solvent and all other reagents were of analytical grade.

### 2.2. Preparation of Cu:ZnO NPs

ZnO and Cu:ZnO NPs were synthesized by co-precipitation. Initially, the 0.5 M solution of zinc nitrate hexahydrate in ethanol was prepared under continuous and vigorous (magnetic) stirring for 10 min at 55 °C, over which (for Cu:ZnO NPs) different atomic concentrations of trihydrate copper nitrate (0.1 at.%; 0.5 at.% or 1 at.%) were added; the resulted solutions were refluxed for about 20 min at 60 °C, until clear solutions were obtained. The potassium hydroxide solution (0.5 M KOH in ethanol) was added dropwise into the as-prepared mixture until some milky white precipitates with a pH of 10 were obtained, indicating the formation of Cu-doped zinc oxide nanoparticles. For a complete precipitation, the mixtures were refluxed for three hours. After completing the precipitation procedure, the obtained powders (ZnO and Cu:ZnO NPs, according to Table 1) were washed with pure water, until achieving a neutral pH, followed by ethanol, and maintained at 80 °C for six hours.

### 2.3. Preparation of MO/MC Hybrid NPs and Coatings

Once the Cu:ZnO NPs, with three different atomic percentages of copper, have been synthesized, they were ready to be incorporated into the MC matrix. The MC was dissolved previously in deionized water to obtain a 0.5 M solution, in which the undoped and Cu-doped ZnO NPs were dispersed. To prevent the agglomeration and ensure a good dispersion in the microcellulose matrix, the ZnO-based NPs were previously dispersed in deionized water by ultrasonication until homogenized and then gradually introduced into the cellulose solution. Then, the GPTMS functionalization agent was added under magnetic stirring and maintained for three hours at ambient temperature. The as-prepared colloidal solutions were introduced into the ultrasonic bath for one hour. After this step, these dispersions were used for coatings deposition onto the textile fibers (Table 2).

MO/MC colloidal solutions prepared as mentioned before (Table 2) were applied as liquid coating agents on textiles (polyester/cotton–50%/50%) by immersion technique and dried at moderate temperatures in an oven (120 °C). After the thermal treatment, and before any characterization, the stabilized coated textile samples were thoroughly washed (US bath) with distilled water heated to 40 °C, during three washing cycles for 2 min/cycle, to remove large, agglomerated particles or formed clusters.

### 2.4. Characterization Methods

X-Ray Diffraction measurements (XRD) of the prepared samples were performed at room temperature, within [10–100]° 2 theta range, by a SmartLab X-Ray Diffractometer (Rigaku Corporation, Tokyo, Japan), using a Cu Ka radiation (λ = 1.542 Å) at 45 kV and 200 mA. The crystalline phases were identified according to the International Center for Diffraction Data—ICDD database.

The morphological properties (shape, size, etc.) of the hybrid nanoparticles were investigated using a Hitachi SU 8230 Scanning Electron Microscope (SEM, Hitachi High-Tech Corporation, Tokyo, Japan), equipped with an Energy Dispersive X-Ray Analyzer (EDAX, Oxford Instuments, Oxford, UK). The morphologies of the hybrid nanoparticles coated textiles were studied by a Quanta 200 Environmental Scanning Electron Microscope (ESEM, former FEI, now Thermo Fisher Scientific, Waltham, MA, USA). Before analysis, the textiles were metallized with conductive layers using cathodic spray—SPI—Etch Mode Module (SPI Supplies, West Chester, PA 19380-4512, USA).

Specific surface areas (S_BET_) were measured by nitrogen physisorption at 77 K using a Micromeritics ASAP 2020 automated gas adsorption system (Norcross, GA, USA). Samples were degassed at 70 °C for 8 h under vacuum before analysis. S_BET_ values were calculated according to the Brunauer-Emmett-Teller (BET) equation, using adsorption data in the relative pressure range between 0.05 and 0.30.

The Raman studies of ZnO and Cu doped ZnO NPs and MO/MC hybrids were carried out at room temperature by confocal µRaman Spectroscopy, using a LabRam HR800 system (Horiba, Kyoto, Japan). All the Raman spectra were generated by exposing the specimens during 900 s to a 1 mW, 532 nm wavelength green excitation laser and dispersing the emitted signal onto the CCD detector using a 600 lines/mm grating with a spectral resolution of 0.6 cm^−1^.

The chemical structure of the prepared samples was studied by Attenuated Total Reflection—Fourier Transform Infrared (ATR-FTIR) spectroscopy, in the 4000–400 cm^−1^ range, using a Thermo Scientific Nicolet iS50 Spectrometer(Madison, WI, USA) equipped with a ZnSe crystal.

The antimicrobial activity of the hybrid coatings deposed on the textile materials was evaluated by the paper disk method on Mueller-Hinton agar against both Gram-negative (*Escherichia coli/E. coli*) and Gram-positive (*Staphylococcus aureus/S. aureus* bacteria). The tests were performed on sterilized disks with a diameter of 6 mm impregnated with 5 µL of prepared sols, which ensured in each sample an amount of active substance (Cu:ZnO) similar to the average concentration of the active ingredients (5 mg/1 mL) from antibiotics used in antibiogram test. 0.2 mL of broth culture incubated for 24 h and 20 mL of melted agar medium were added to each impregnated disk, at about 50 °C. After cooling to ambient temperature, each disk was placed in contact with the bacteria-inoculated agar and maintained in an incubator at a constant temperature of 37 °C ± 1 °C for 24 h, according to the standardized antimicrobial testing method used.

The optical band gap energy, E_g_, of the Cu-doped ZnO NPs dispersed in ethylic alcohol and deposited as thin films on Soda lima glass substrates by spin-coating technique (at 2000 rpm) was estimated from the fundamental absorption edge in their optical transmittance and reflectance spectra, acquired in air at room temperature with a Perkin Elmer Lambda 35 spectrometer, at normal incidence, in the 200–1100 nm spectral range. For E_g_ evaluation, the absorption coefficient was calculated using the following Equation (1) [40]:

(1) $$\alpha =\frac{1}{d}\ln\left[\frac{{\left(1-R\right)}^{2}+\sqrt{{\left(1-R\right)}^{2}+4R^{2}T^{2}}}{2T}\right]$$

where d is the film thickness, T is the transmittance and R reflectance of the ZnO 1D nanostructured thin films. The E_g_ values were calculated from the dependence of absorption coefficient vs. the photon energy, hν, based on the following Equation (2) [19,20,21,41]:

(2) $${\left(\alpha h\nu \right)}^{2}=A(h\nu -E_{g})$$

where A is a parameter that depends on the transition probability.

The photocatalytic activity of the obtained hybrid nanoparticles and textile coatings was investigated by measuring the photodegradation of Methylene Blue (MB) and Methylene Orange (MO) aqueous solutions (20 mg/L), under ultraviolet irradiation (254 nm). The absorption spectra of MB and MO solutions after 1 or 2 h irradiation were recorded in the Visible (400–800 nm) range, using a Microplate reader with fluorescence spectrometer Infinite 200 PRO NanoQuant (Tecan, Switzerland).

## 3. Results

**Figure 1:**
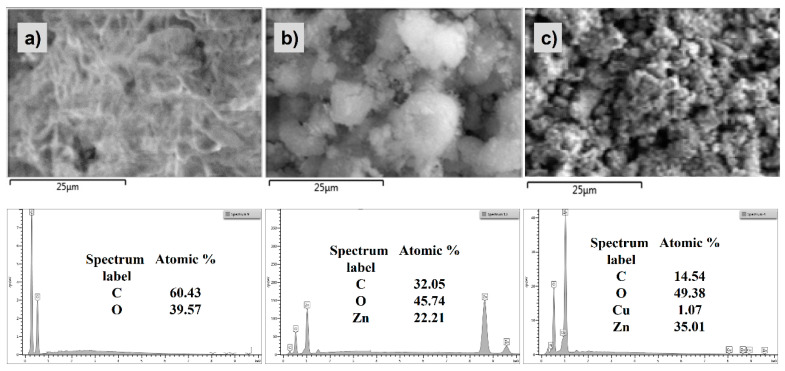
SEM micrographs revealed by: (**a**) MC, (**b**) ZnO/MC and (**c**) Cu (1 at.%):ZnO/MC samples, together with their corresponding EDX spectra.

Figure 1 shows the SEM micrographs, revealed by MC, ZnO/MC and Cu:ZnO/MC-3 NPs samples, together with their corresponding EDX elemental analysis. As can be observed, the morphology of microcellulose, which exhibits a dense and porous network structure (Figure 1a), changes significantly after the incorporation of the undoped ZnO nanoparticles, showing a strong tendency to aggregate, forming large and irregularly shaped clusters, that are leading to a considerable increase in the surface roughness (Figure 1b). The 1 at.% Cu doped ZnO/MC hybrid sample shows a significantly different morphology, defined by nanoparticles with smaller sizes, which, as expected, are leading to an increase in the density of the pores (Figure 1c).

The EDX spectra unveil the presence of C and O chemical elements in each studied sample, both specific for the microcellulose matrix. The successful incorporation of the ZnO nanoparticles into the prepared hybrids, ZnO/MC and Cu:ZnO/MC, is evidenced by the results obtained from the performed elemental analysis, which clearly confirms the presence of both, Zn and O, elements. Furthermore, the EDX analysis certifies the Cu doping of the Cu:ZnO/MC-3 sample, validating also the added Cu atomic percentage (1 at.%).

**Figure 2:**
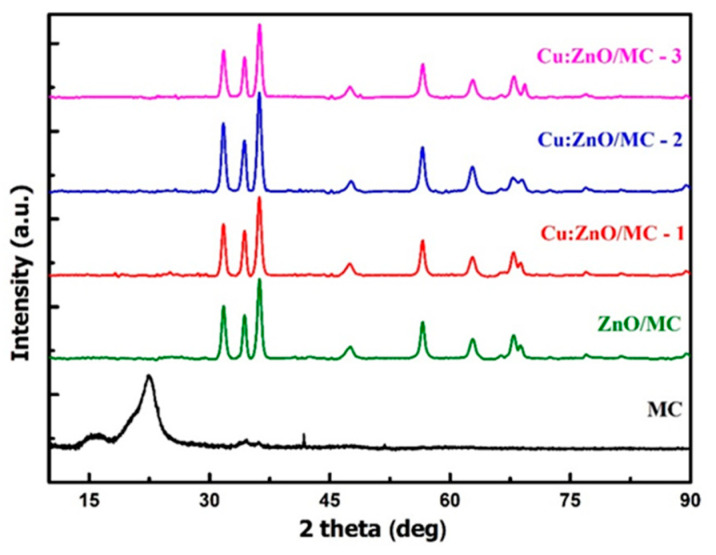
XRD patterns recorded for MC, ZnO/MC, Cu (0.1%):ZnO/MC, Cu (0.5%):ZnO/MC and Cu (1%):ZnO/MC samples.

Figure 2 presents the X-Ray diffraction patterns, reveled by the MC, ZnO/MC and Cu doped (0.1 at.%, 0.5 at.% and 1 at.%) ZnO/MC nanoparticle samples. The XRD diffractogram recorded for MC is defined by three main reflections at 16.30, 22.40 and 34.40 2theta angles, assigned to 110, 200 and 400 planes, which are characteristics for the crystalline cellulose (I) [42]. The diffraction peaks observed for the undoped hybrid nanoparticles (ZnO) at 31.74, 34.38, 36.22, 47.54, 56.62, 62.75, 67.85, 68.68, 76.82 and 81.23 2theta angles, corresponding to 100, 002, 101, 102, 110, 103, 112, 202, 104 and 203 crystalline orientations, respectively, are assigned to the ZnO phase having a hexagonal crystal system within P63mc space group, using the reference 01-075-6445 JCPDS card. The XRD pattern revealed by the ZnO phase is in good agreement with other results reported in the literature [43,44,45]. As can be seen, the diffraction peaks exhibited by the hybrids are notably sharp and intense, suggesting a high crystallinity degree of the investigated samples. The diffraction patterns of the Cu-doped hybrid nanoparticles are highly similar to the one exhibited by the undoped sample. No clear phase changes were observed, as a result of the Cu addition. As suggested in [46], this behavior may indicate that the Cu ions have replaced the Zn sites, without a noticeable influence on the ZnO structure, most likely forming a new phase, copper zinc oxide ((Zn_0.9_Cu_0.1_)O—01-081-9217 JPCDS card), which also has a hexagonal crystal system, but smaller cell parameters and, consequently, a slightly smaller volume which could be correlated to the size reduction in the NPs with the addition of Cu content. The formation of the (Zn_0.9_Cu_0.1_)O phase as a consequence of Cu doping is also supported by the EDX results which clearly confirm the presence of the Cu in doped hybrid NPs (Figure 1).

**Figure 3:**
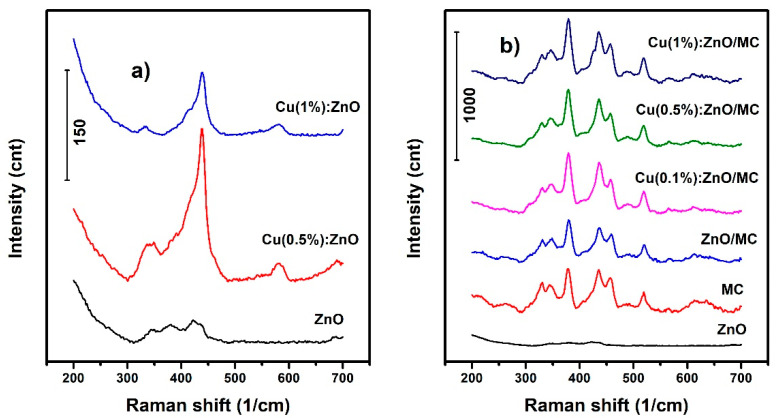
Raman spectra recorded for: (**a**) starting oxide NPs (ZnO and Cu doped ZnO) and (**b**) MC and hybrid NPs (ZnO/MC and Cu doped ZnO/MC) samples.

Figure 3a presents the Raman spectra revealed by the undoped and Cu doped ZnO NPs within 200–700 cm^−1^ spectral range. The positions, widths and intensities of the peaks were precisely identified by applying spectra deconvolutions (see Supplementary, Figure S1) using Lorentz/Gauss functions, after performing a spline baseline correction. The Raman spectrum of ZnO was deconvoluted to 4 bands at 344.7, 378.9, 424.3 and 438.3 cm^−1^, corresponding to the E_2_(high)–E_2_(low), A_1_(TO), E_1_(TO) and E_2_(high) modes, typical for hexagonal wurtzite phase [47]. All these peaks are also exhibited by the Cu doped NPs, but at different positions. The bands generated by E_2_(high)–E_2_(low) and E_1_(TO) processes are redshifted by ~5/15 and 3/6 cm^−1^, while the A_1_(TO) band experiences a blueshift with ~5/7 cm^−1^ for Cu (0.5%):ZnO and Cu (1%):ZnO samples, respectively. The E_2_(high) band that usually appears in ZnO due to the oxygen atoms vibrations, undergoes a very small blueshift of 0.5/07 cm^−1^ for Cu doped NPs, which can be ignored as these variations are almost in system’s resolution limit, but the significant increase in intensity as well as band sharpening should be noted. Similar studies in the literature reported an opposite behavior for intensity, correlating it with a reduction in the crystallinity of the materials [48,49]. In our samples, the degree of crystallinity, implicitly the hexagonal structure, is preserved and even improved after Cu doping, as shown also by the XRD results. The reduction in the lattice disorder [50] is also suggested by the noticeable sharpening of the bands in Cu doped NPs compared with the broad one revealed by ZnO NPs. It should be mentioned that the sample doped with 1 at.% Cu showed a decrease in the intensity of the E_2_(high) band compared with the sample doped with 0.5 at.%. Also, after Cu doping, the appearance of a new la band at ~581 cm^−1^ can be observed, which can be attributed to B_1_(high) silent mode, that can suggest the presence of some defects/impurities which might have been generated by the process of replacement of Zn ions by Cu ions, thus confirming the successful Cu doping in ZnO lattice [51].

Figure 3b shows the Raman signature of hybrid NPs, along with the ones exhibited by the starting materials used for their preparation, MC and ZnO. As can be seen, the Raman fingerprint of MC is defined by a multi-peaked profile over 300–550 cm^−1^ spectral range. There are no easily discernible differences between the spectra exhibited by the hybrids compared to the one of MC, unsurprising behavior considering that their much less intense undoped and Cu-doped ZnO peaks overlap/are covered by the MC bands. However, by deconvolution of the MC and MC based samples bands (hybrids), differences were identified in their positions, the most significant being reveled by the MC peak at 417 cm^−1^ which suffered a redshift of 3/3/7 cm^−1^ for the hybrids with 0, 0.1 and 0.5 at.% Cu, and a blueshift of 6 cm^−1^ for the one doped with 1 at.% Cu. The shift in the specific MC bands confirms the presence of the undoped and doped ZnO in the investigated samples.

**Figure 4:**
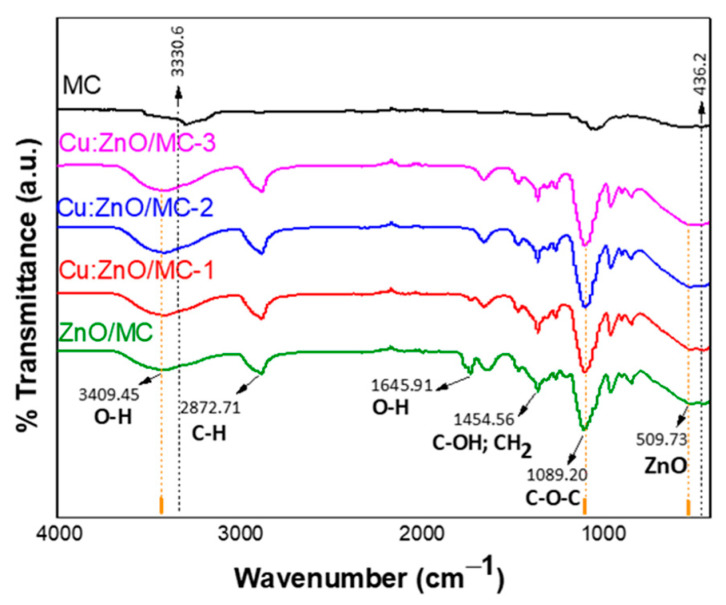
ATR-FTIR spectra of MC, ZnO/MC, Cu (0.1%):ZnO/MC, Cu (0.5%):ZnO/MC and Cu (1%):ZnO/MC samples.

The ATR-FTIR spectra of the MC and ZnO-based hybrid nanoparticles are illustrated in Figure 4. At first observation, all the recorded FTIR spectra reveal the presence of a representative pattern of cellulose, with some relevant changes between samples.

The wide peak at 3409 cm^−1^ and the one from 1645 cm^−1^ correspond to bending vibrations of the surface O-H groups, the latter being specific to adsorbed water [52]. The splitting of the last band was observed for all the samples, including Cu:ZnO NPs, and the intensity of the resulting bands increased with the Cu-doping up to 1 at.%. This confirms the increased amount of adsorbed water and water-cellulose interaction when their size decreases (Figure 1c). At the same time, the higher intensities of 3409 cm^−1^ for ZnO/MC and Cu:ZnO/MC samples, when compared with the peak of pure cellulose (3350 cm^−1^), clearly indicates the presence of direct interactions between these nanoparticles and the surface OH groups of cellulose. Peaks at 2872 and 1367 cm^−1^ are assigned to the stretching and bending vibrations of C-H, while the CH_2_ bending vibration of the pyranose ring of glucose can be observed at 1454 cm^−1^. The most intense and sharp peak from 1089 cm^−1^ with a shoulder at 1050 cm^−1^ is attributed to C-O-C vibrational pyranose ring of cellulose [53,54]. The presence of Zn-O vibration around 509 cm^−1^ (Figure 4) indicates the existence of ZnO [55]. For ZnO covered with cellulose, peaks at ~420 and 510 cm^−1^ were reported [56]. The peak at 509 cm^−1^ overlaps with the cellulose peak at ~550 cm^−1^ [53]. As detailed in the Section 3. Results (Figure 4) and according to Table S2 (see Supplementary), peaks at different frequencies and shifts of the absorption bands from the FTIR spectra can be observed for the samples containing ZnO/MC, Cu (0.1%):ZnO/MC, Cu (0.5%):ZnO/MC and Cu (1%):ZnO/MC samples, compared with the MC (microcellulose) sample, confirming the presence of ZnO and its effective interaction with MC molecules.

**Figure 5:**
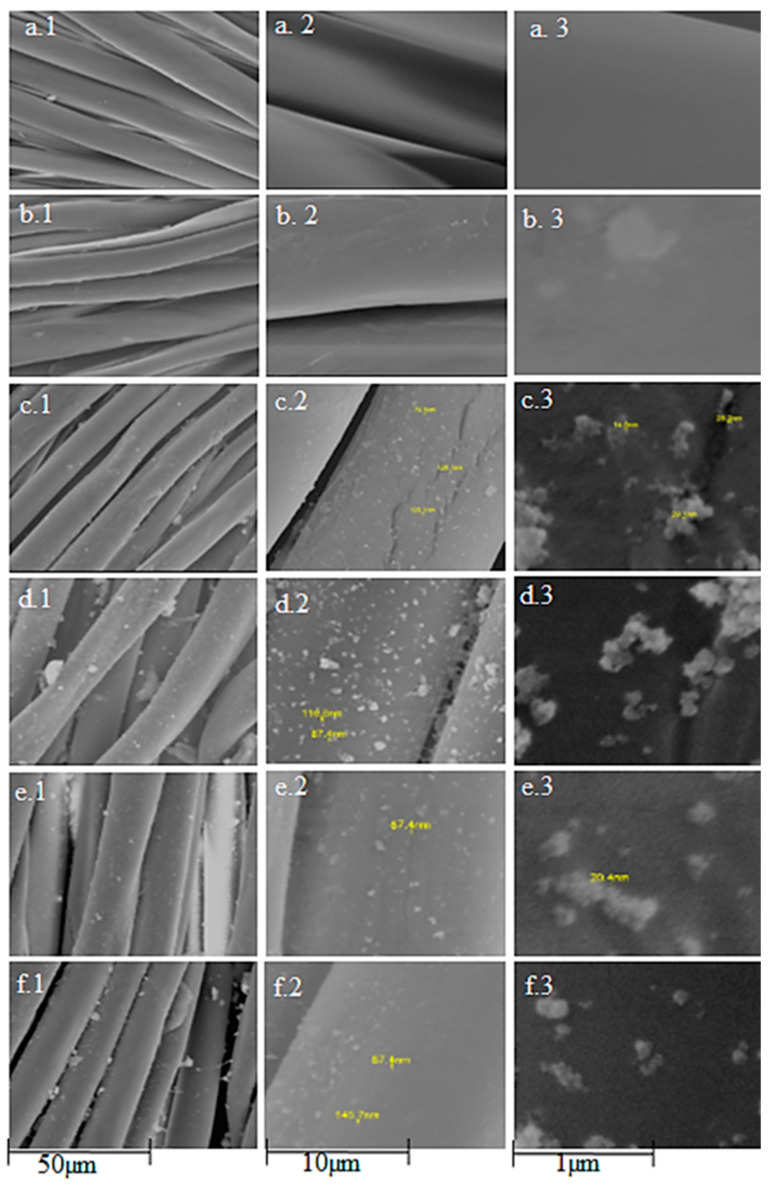
SEM micrographs of the polyester/cotton mixture (50%/50%): untreated (**a.1**–**a.3**) and coated with MC (**b.1**–**b.3**), ZnO/MC (**c.1**–**c.3**), Cu (0.1%):ZnO/MC (**d.1**–**d.3**), Cu (0.5%):ZnO/MC (**e.1**–**e.3**), Cu (1%):ZnO/MC (**f.1**–**f.3**), recorded at different magnifications.

Figure 5 presents the SEM micrographs of the uncoated and MC, ZnO/MC and Cu doped ZnO/MC coated polyester/cotton (50%/50%) fabrics. As expected, the untreated fiber’s surface is smoother (Figure 5a) compared with those exhibited by the MC and MO/CM hybrids coated fabrics, which show rougher surface profiles. It should be noted that the MC-coated textile surface (Figure 5b) shows large microcrystallite islands, which are different from the aggregates exhibited by the ZnO/MC-covered fabric (Figure 5c). The influence of the Cu-doping concentration on the morphology of the hybrid Cu:ZnO/MC coatings illustrated by SEM analysis (Figure 5d–f), is confirmed by the smaller sizes of NPs and a smoother surface for Cu (1 at.%)ZnO/MC coating (Figure 5f). As mentioned before, the size of the nanoparticles plays an important role in the adhesive properties of the textile fibers. The larger particles or clusters are expected to be more easily removed from the surface of the fibers after deposition, while the small-sized particles show greater probability of penetrating (deeper) into the pores and intermolecular spaces of the fibers, thus leading to an increase in surfaces’ adherence and durability.

**Figure 6:**
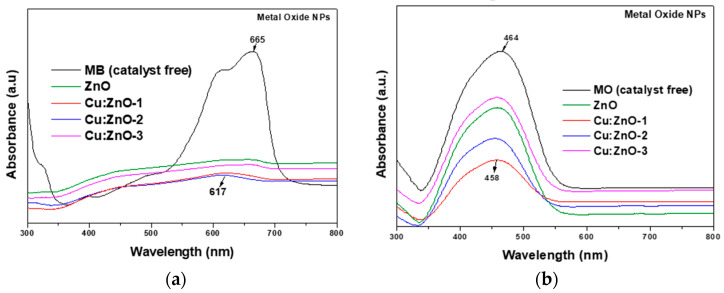
UV-Vis absorption spectra of photodegraded MB (**a**) and MO (**b**) solutions after up to 120 min UV irradiation in the presence of ZnO and Cu-doped ZnO NPs.

The photocatalytic activity of the investigated (Cu)-doped ZnO NPs and corresponding hybrid coatings with different dopant concentrations was tested by the degradation of MB and MO dyes under one and two hours of UV irradiation, which is directly proportional to the decrease in the measured optical absorbance of MB or MO dye solutions, the most intense peaks at 663 and 463 nm, respectively, were used. Figure 6 illustrates the UV-Vis absorption spectra of photodegraded MB (a) and MO (b) solutions after up to 2 h UV irradiation in the presence of ZnO and Cu-doped ZnO NPs.

**Figure 7:**
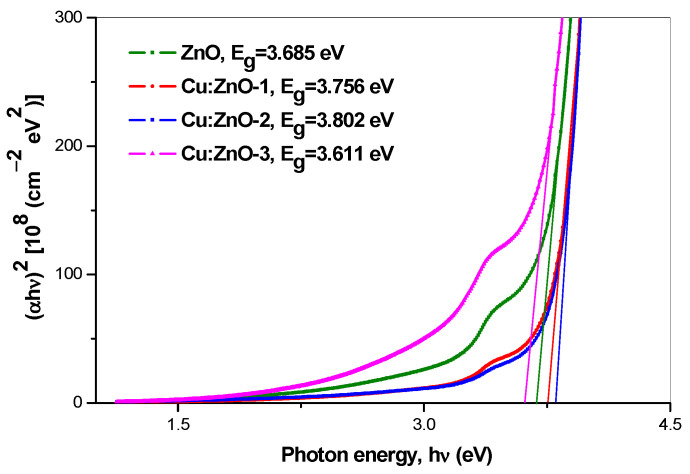
Plot of (αhν)^2^ vs. hν for ZnO and Cu:ZnO NPs.

Analyzing the photocatalytic behavior of simple oxide NPs obtained by (co)precipitation, firstly, a consistently better activity regarding the degradation of MB (Figure 6a) compared with that for MO (Figure 6b) is highlighted. Secondly, it is observed that in both cases (MB and MO) there is no monotonous variation of the catalytic activity with increasing Cu-doping between 0.1 and 1 at.%. Thus, for both dyes, NPs doped with 1 at.% Cu have a behavior quite close to undoped ZnO NPs, while NPs doped with 0.1 and 0.5 at.% Cu seem to have a somewhat better photocatalytic activity. To explain this behavior, since it is about photocatalysis with nanostructured semiconductor catalysts, the band gap energy was calculated from the plots against the photon energy, hν. The optical band gap energy E_g_ values, determined by extrapolating the linear portion of the curves to the *hν* axis, are shown in Figure 7. First, a higher E_g_ value of 3.685 eV can be observed for the undoped ZnO NPs compared with the bulk E_g_ value (3.2–3.3 eV) [41]. Then, it is shown that with doping, the optical E_g_ increases steadily to 3.756 (0.1 at.% Cu) and 3.802 (0.5 at.% Cu), to then drop to 3.611 eV when the Cu doping concentration increases to 1 at.%. This behavior correlates well with the variation of the intensity absorption peaks for the photodegradation of dyes (Figure 6), especially in the case of MO (Figure 6b).

If in the case of NPs doped with 0.1 and 0.5 at.% the increase in E_g_ can be attributed to the quantum size effect (the blueshift effect when the NPs size decreases by doping in the range below 10 nm/quantum dots), the behavior of the sample with 1 at.% needs further investigation. In this context, it is interesting to note that this trend of band gap energy variation with increasing Cu-doping can be also observed in the variation of the intensity of the XRD (Figure 3) and Raman (Figure 4) peaks. Thus, for the samples containing zinc oxide doped with 1 at.%, a decrease in the intensity of these peaks is observed, compared with the case of the samples in which the doping was only 0.1 and 0.5 at.% Cu. In conclusion, it could be considered that although there are no evident phase separations in XRD and Raman data, the increase in Cu content up to 1 at.% produces significant changes in the energy bands of the materials.

**Figure 8:**
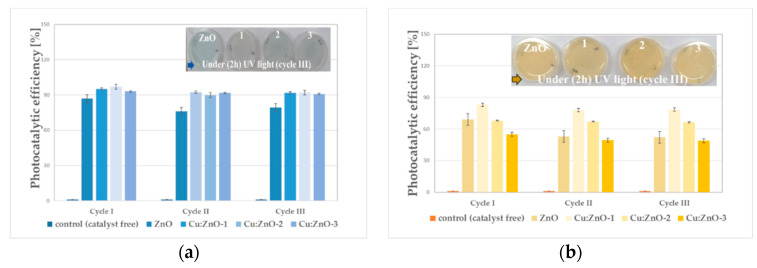
Photodegradation efficiencies of the NPs catalysts for MB (**a**) and MO (**b**) dyes degradation under UV irradiation (120 min), reutilized for three consecutive cycles.

The photocatalytic efficiency of the NPs during three cycles of MB and MO dyes degradation, calculated according to the Equation (3) [57], is presented in Figure 8:

(3) $$\eta =\frac{C_{0}-C_{t}}{C_{0}}\times 100=\frac{A_{0}-A}{A_{0}}\times 100$$

where $C_{0}$ is the initial concentration of the dye, $C_{t}$ is the concentration of the dye at time ‘*t*’, $A_{0}$ and A are the corresponding absorbance values of the concentrations $C_{0}$ and $C_{t}$, respectively.

The photodegradation efficiency increases for all Cu-doped ZnO NPs. (Figure 8). A different behavior can be observed with increasing doping concentration for the degradation of the two dyes. Thus, MB degradation increases/changes slightly/insignificantly with Cu-doping until 1 at.% (Figure 8a), while it decreases for MO degradation (Figure 8b). Overall, the results show, for both MB and MO dyes, a good stability concerning the photocatalytic efficiency during reuse for three cycles.

**Figure 9:**
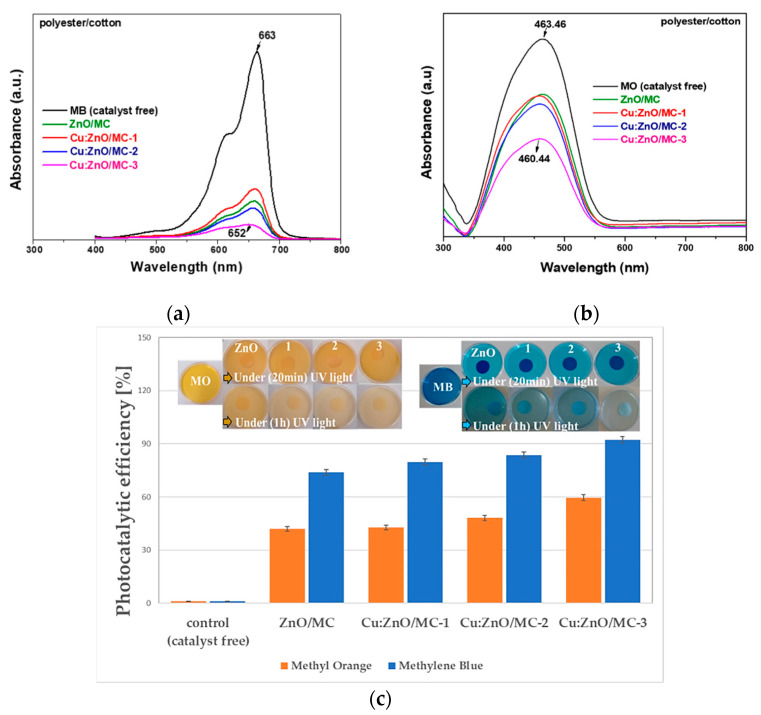
The photocatalytic activity of uncoated, ZnO/MC and Cu-doped ZnO/MC coated polyester/cotton (50%/50%) for methylene blue (MB) and MO aqueous solutions degradation: the optical absorption spectra (**a**,**b**) and photocatalytic efficiency (**c**).

Figure 9 shows the optical absorption spectra of aqueous solutions of MB and MO, recorded in the presence of ZnO/MC and Cu-doped ZnO/MC coated polyester/cotton (50%/50%) textiles. In the first 20 min, the MB adsorption on the surface of the hybrid nanoparticles deposited on the textile material disk (see detail in Figure 9c) was observed, where the redox reactions occur.

Figure 9 shows a significantly different photocatalytic behavior of the hybrid coatings compared with the starting oxide NPs (Figure 8a,b) used for coating preparation. A continuously increasing trend of photocatalytic efficiency was observed, both for MB and MO degradation (Figure 9c) with increasing the Cu-doping concentration. This behavior can be attributed to the dispersion and stabilization of oxide NPs at the nano-metric scale following the interaction with MC and the surfactant effect of the cross-linking agent (GPTMS). In common with the oxide nanoparticles (Figure 8b), the hybrid coatings show significantly lower photocatalytic efficiency for MO degradation (Figure 9c).

In the case of MB degradation, the photocatalytic efficiency of the investigated hybrid coatings increases from 78.6 to 91.7%, with the increase in Cu-doping from 0.1 to 1 at.%, while for MO degradation this increase is only from 42.7 to 59.6% (Figure 9c).

These different photocatalytic behaviors of the uncoated and MC-coated Cu-doped ZnO NPs seems to correlate with variation on the BET surface area (S_BET_) only in the first case (Figure 8), and especially for MO degradation (Figure 8b). From the analysis of the data presented in Table S1 (see Supplementary), it follows that the S_BET_ values increase/double (from 2.8 to 6.4 (m^2^/g), as expected, by doping ZnO with 1 at.% Cu. The S_BET_ value also increases, from 2.8 to 3.6 g/m^2^, for undoped ZnO NPs incorporated into MC. Unexpectedly, the behavior of Cu-doped NPs changes following their incorporation into microcellulose. Thus, S_BET_ increased to 9.3 for Cu (0.1 at.%)%) ZnO/MC and it decreased to 4.3 for Cu (1 at.%) ZnO/MC NPs. In these conditions, the increase in photocatalytic and antimicrobial efficiency can no longer be attributed to the increase in the specific surface area, as it happens in the case of simple oxides NPs, the interaction with MC and GPTMS cross-linking agent being probably responsible for these behaviors.

**Figure 10:**
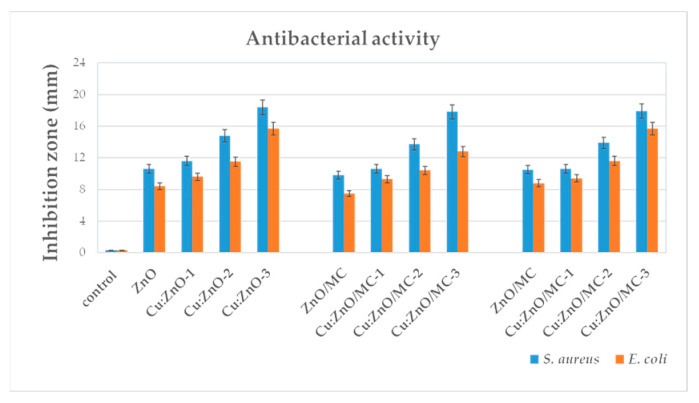
Antibacterial activity of undoped ZnO, Cu:ZnO and Cu:ZnO/MC (different Cu-doping) NPs and coatings on textile fibres, against *S. aureus* and *E. coli* bacteria.

The results of the antimicrobial test performed on the hybrid nanoparticles and corresponding coatings deposed on the textile materials against both Gram-negative (*Escherichia coli/E. coli*) and Gram-positive (*Staphylococcus aureus/S. aureus* bacteria) are shown in Figure 10.

The diameter of the inhibition zone increases from 10 to 18 mm both for Cu:ZnO oxide NPs, Cu:ZnO/MC hybrid NPs and for Cu:ZnO/MC/Textile hybrid coatings, in the case of *S. aureus* bacteria, and only up to ~16 mm for *E. coli*, when the doping is 1 at.% Cu. These results indicate a stronger activity on gram-positive *S. aureus* bacteria.

## 4. Discussion

Semiconductive MO-based hybrids bring two significant advantages when used as multifunctional coatings, namely self-cleaning (based on UV photocatalytic effect and UV blocking) (1) and substitute of antibiotic, without microbes’ resistance developed by antibiotics, when used as an antimicrobial agent (2). Combining MO semiconductors with MC brings multiple advantages, such as high dispersibility and stability of MO, increased density and stability of cellulose nanocrystals and biocompatibility, biodegradability and tuned electrical conductivity of the final hybrid material.

Finding cost-effective and efficient methods using MO-polymers hybrid composites to achieve adherent, cost-effective, and efficient multifunctional innovative coatings on textiles, smart packaging, hospital fabrics and on various surfaces of materials and objects from public or sanitary environments is a major desideratum.

A simple new method was developed in this study to prepare Cu-doped ZnO/MC hybrid multifunctional coatings to be deposed as a liquid water-based agent onto natural and/or synthetic fibers in the presence of GPTMS cross-linking agent. Hybrid nanoparticles and corresponding coatings on polyester/cotton–50%/50% fibers were obtained. The formation of different morphologies of Cu-doped ZnO/MC nanoparticles and coatings and their photocatalytic and antimicrobial activity are discussed in terms of Cu-doping concentration, which varied from 0 (pure ZnO NPs) to max. 1 at.%.

When compared with the monocomponent original MC sample with fibrous morphology (Figure 1), the undoped ZnO/MC sample shows a macroporous globular texture [58], consisting of a majority component of cellulose microparticles (5–15 μm) and dispersed submicron ZnO particles deposited on the surface or at the intergranular boundaries of the former. The majority cellulose phase is confirmed by the elemental composition (C:Zn ~ 32:22 at.%) indicated by the SEM-EDX data in Figure 1. XRD results highlight the reduction in the ordering degree of MC from long-order to as short-order arrangement when adding oxide nanoparticles. The lack of the diffraction peaks of type I crystalline structure of cellulose (2theta between 15–25°) [42], present in the original MC sample used for the preparation of hybrid nanoparticles (MC in Figure 2), indicates, most likely, the transformation of the cellulose’s structure present into the hybrid composite (Figure 2) into a quasi-amorphous.

**Figure 11:**
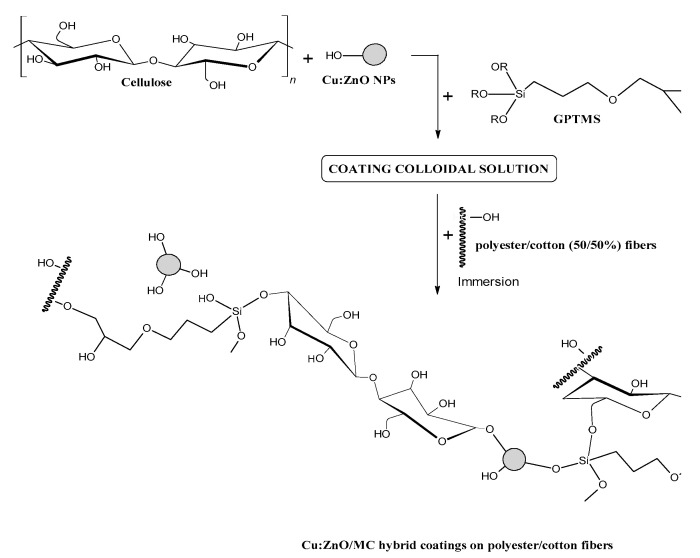
The proposed interactions during the syntesis of MO/CM colloidal solutions and corresponding coatings deposed on textile fibres, in the presence of GPTMS cross-linking agent.

The top-view SEM image indicates for the Cu-doped hybrid nanoparticles a fine and homogeneous globular structure with spherical particles of sizes between 500–800 μm, assembled in a microporous texture (Cu(1 at.%):ZnO in Figure 1). The consistent increase (more than 2.5 times) in the concentration of Zn in the sample doped with 1 at.% Cu compared with the non-doped one (EDX Specter in Figure 1), confirms a very good dispersion and inclusion of Cu (1 at.%):ZnO nanoparticles in a minority cellulose network. The value of 1:2.4 of the [C]:[Zn] ratio (EDX spectra in Figure 1) indicates not only a breaking of the intermolecular bonds between the cellulose chains that led to the transformation/disappearance of the globular micronic cellulose formations (for ZnO/MC) but also of the intramolecular ones with a fragmentation of the latter, caused by strong interactions with the doped nanoparticles (Cu:ZnO). These interactions were favored by reducing their size with Cu-doping (Figure 1c), and on the other hand by the presence of GPTMS molecules that ensure the cross-linking between ZnO and the cellulose chains. Figure 11 proposes a mechanism of interactions between components during the preparation of the hybrid MO/MC NPs and corresponding coatings.

During cellulose dissolving and MONPs dispersion in water, a significant number of water molecules are fixed on the surface of the nanoparticles by adsorption or chemo-sorption. A complex heterogeneous colloidal system is generated by adding the aqueous dispersion of MONPs to the aqueous solution of cellulose under US stirring. When GPTMS is added to this colloidal dispersion of MO/MC (Figure 11), after the hydrolysis of the silane molecules, and during the US sonication, competition in the formation of various bridges by condensation reactions, including complex ones as (MC)-C-O-Si-O-ZnO, can be generated. For the undoped ZnO/MC hybrid composite nanoparticles formation, a phase separation mechanism can be considered, namely the formation of a micronic globular phase of cellulose and a submicron phase of ZnO, connected to each other at the interface by the cross-linking agent, resulting a heterogeneous composite (Figure 1b). In the case of Cu:ZnO/MC samples, the results indicate the formation of real hybrid nanoparticles, very homogeneous morphologically (Figure 1c) due to the compositional and structural homogeneity based on interactions at the molecular level between the component entities (Figure 11). Advanced structural studies will be performed to elucidate the chemical structure of the hybrids formed with Cu:ZnO doped-NPs, exploring a more comprehensive range of doping concentrations (up to 5 at.%), but also of Cu:ZnO/MC/GPTMS molar ratios, corroborated with photocatalytic efficiency and antimicrobial. Another direction of research to complete this study is the investigation of the stability of the NPs and the coatings with the highlighting of the photocatalytic efficiency in more reuse cycles. The study is also open to testing the method for depositing various Metal Oxide/MC type hybrid coatings on different types of surfaces.

As mentioned before, the photocatalytic and antimicrobial activities of Metal Oxide-based nanomaterials are generally explained based on the presence of reactive oxygen species (ROS) responsible for producing oxidative stress on the bacteria [59,60,61,62,63,64]. Recently was demonstrated that part of the antibacterial activity of ZnO is determined by its ability to mimic biological inhibitors by matching their geometry with that of protein-based inhibitors [55]. The increased photocatalytic efficiency of ZnO/MC nanoparticles with Cu-doping (up to 15%) was reported by other authors [48,59,60,61,62,63,64,65]. The explanation of the enhanced photocatalytic efficiency of Cu–doped ZnO nanoparticles differs depending on the doping concentration. Thus, for doping below 2%, the improvement of photogenerated charge carriers’ separation is achieved through an efficient substitution of Cu ions into the ZnO crystalline network [59], while at higher dopings it is about the formation of ZnO–CuO nano-heterojunctions [48]. The related mechanisms are presented in [48,59]. Compared with these studies, our results highlighted that an only 1 at.% Cu-doping leads to a better effect on MB degradation efficiency of 92% after 60 min UV irradiation, including in the case of Cu-ZnO/MC composite nanoparticle deposition on the cellulose:polyester (1:1) textile. The UV-Vis absorption spectra of photodegraded MB and MO solutions (Figure 6 and Figure 9) show not only a decrease in the absorbance peak intensity by increasing Cu-doping concentration from 0 to 1 at.%, but also blue shifts from 663 to 651 nm (Figure 6a) and 467–458 nm (Figure 6b), confirming the effect of successful incorporation of the Cu atoms into the crystalline structure of ZnO nanoparticles on their electronic band structure. The antibacterial activity of the investigated samples increases as the size of the nanoparticles decreases (Figure 10). ZnO demonstrated a synergic antibacterial effect on Gram-positive bacteria [49]. For our study, the best inhibition activity was revealed by MONPs doped with 1 at.% Cu. The dispersion of these MONPs into cellulose matrix determined to a very small decrease (case Cu(1 at.%)ZnO/MC NPs), but it remained significantly better than for the undoped or doped samples below 1 at.% Cu. It is relevant to highlight that Cu(1 at.%)ZnO/MC textile coatings have a higher activity than NPs with the same composition and are similar to that of Cu(1 at.%)ZnO NPs. Also, the antibacterial textiles coatings (Figure 10) highlighted a stronger antibacterial activity against the higher resistant *S. aureus* Gram-positive bacteria, than against gram-negative *E. coli*, which also depends on the size of the Cu doped nanoparticles.

## 5. Conclusions

New Cu-doped ZnO/MC hybrid coatings on textile fibers (polyester/cotton, 50%/50%) have been successfully obtained using a modified sol-gel route assisted by GPTMS organosilane cross-linking agent, from an aqueous solution of microcellulose. To the best of our knowledge, there are only a few published works which report the preparation of cellulose/ZnO-based nanocomposites directly from cellulose aqueous solution. Moreover, direct use of a silane (GPTMS) in the aqueous MC solution containing ZnO-based nanoparticles with very low Cu-doping (0.1–1 at.%) to ensure very good dispersibility of the latter in solution but also for very good adhesion of the hybrid coatings to the textiles, represents the novelty of this study.

The Raman and XRD measurements have confirmed the wurtzite-type crystal hexagonal structure with preferred orientation in 101 crystallographic plane for all the prepared, Cu-doped ZnO NPs and coatings.

The hybrid Cu:ZnO/MC coatings deposited on textile materials and stabilized at moderate temperatures (120 °C) show good dispersion and low nanoparticles aggregation. Cu-doping up to 1 at.% resulted in a drastic decrease in the Cu-doped ZnO/MC NPs size and also in higher morphological uniformity, which led to a consistent increase, approximately 3-times by EDX results, in the number of ZnO-based NPs inside the hybrid coating.

Different photocatalytic behavior of the Cu-doped ZnO NPs for MB (665 nm absorption peak) and MO (464 nm absorption peak) dies degradation was observed. Following our results, it seems that in the first case (MB) the photocatalytic efficiency correlates with the variation of the band gap energy (E_g_) and in the second (MO) with the S_BET_ variation.

Significantly different photocatalytic behavior of the hybrid coatings compared with the starting oxide NPs used for coating preparation was also observed. The reduced MONPs’s size by Cu-doping, the presence of silane cross-linking agent and the ability of micro/nanocellulose surface active sites for heterogeneous nucleation have reduced the NPs’ size and agglomeration, increased their dispersibility and lead to the formation of active photocatalytic and antimicrobial Cu:ZnO/MC coatings. Low (1 at.%) copper-doping has shown a very significant increase in ZnO-based/CM coatings properties. About 92% and 60% photocatalytic efficiency on Methylene Blue and Methylene Orange degradation during 60 min UV irradiation and strong antibacterial activity of about 18 and 16 mm inhibition zone diameter against *S. aureus* and *E. coli*, respectively, were demonstrated.

A mechanism of interactions between components during the preparation of hybrid MO/MC NPs and coatings was attempted. Advanced structural studies will be performed to highlight and confirm the nature of the interactions between the components, given the presence of the GPTMS cross-linking agent.

## Figures and Tables

**Table 1:** Prepared MO sols and NPs.

Sample	[ZnO] (mol/L)	[Cu] (at.%)	Sample Code
ZnO Cu:ZnO	0.035	-	ZnO
		0.1	Cu:ZnO-1
		0.5	Cu:ZnO-2
		1	Cu:ZnO-3

**Table 2:** Prepared MO/MC NPs and coatings.

Sample	[ZnO] (mol/L)	[Cu] (at.%)	Sample Code	
			NPs	Coatings
MC	-	-	MC	MC textile
Cu:ZnO/MC	0.035	-	ZnO/MC	ZnO/MC-textile
		0.1	Cu:ZnO/MC-1	Cu:ZnO/MC-1 textile
		0.5	Cu:ZnO/MC-2	Cu:ZnO/MC-2 textile
		1	Cu:ZnO/MC-3	Cu:ZnO/MC-3 textile

## Data Availability

All the data supporting the findings of this study are available within the article, and in the supplementary.

## Supplementary Materials

The following supporting information can be downloaded at: https://www.mdpi.com/article/10.3390/ma15217656/s1, Figure S1: Representative examples of Raman spectra deconvolution for different samples: ZnO, Cu doped ZnO, ZnO/MC and Cu(1%):ZnO/MC; Table S1: S_BET_ (Brunauer-Emmett-Teller) values of the investigated oxide and hybrid NPs, Table S2: ATR-FTIR frequencies characteristic of the MC and hybrid (ZnO/MC, Cu (0.1%):ZnO/MC, Cu(0.5%):ZnO/MC and Cu (1%):ZnO/MC) NPs.
