# Supplementary material for: Antibacterial and Photocatalytic Coatings Based on Cu-Doped ZnO Nanoparticles into Microcellulose Matrix
Source: Materials (Basel). 2022 Oct 31;15(21):7656. doi: 10.3390/ma15217656 (PMC9655606; doi:10.3390/ma15217656)
Supplement: Supplementary file 1 [file materials-15-07656-s001.zip › materials-1922433-supplementary.pdf]

Supplementary information

Antibacterial and Photocatalytic Coatings based on Cu-doped ZnO Nanoparticles into Microcellulose Matrix

Mariana Busilă[1+], Viorica Mușat[1*], Rodica Dinică[2], Dana Tutunaru[3], Aida Pantazi[4], Dorel Dorobantu[4,5], Daniela C. CULIȚĂ[6], Marius Enăchescu[4,7*]

[1]LNC-CNMF - Center of Nanostructures and Functional Materials, Faculty of Engineering, ”Dunărea de Jos” University of Galati, 111 Domneasca Street, 800201, Galați, Romania

[2]Department of Chemistry, Physics and Environment, Faculty of Sciences and Environment, ”Dunărea de Jos” University of Galati

[3]Faculty of Medicine and Pharmacy, ”Dunărea de Jos” University of Galati, Street, 800, Galați, Romania

[4]**CSSNT -** Center for Surface Science and Nanotechnology, University Politehnica of Bucharest, 313 Splaiul Independentei, 060042, Bucharest, Romania

[5]S.C. NanoPRO START MC S.R.L., Mitropolit Antim Ivireanu Street 40, 110310 Pitesti, Romania

[6]Institute of Physical Chemistry "Ilie Murgulescu" of Romanian Academy, Bucharest, Romania

[7]Academy of Romanian Scientists, Splaiul Independentei 54, 050094, Bucharest, Romania

* Correspondence: authors: vmusat@ugal.ro (V.M.), marius.enachescu@cssnt-upb.ro (M.E.)

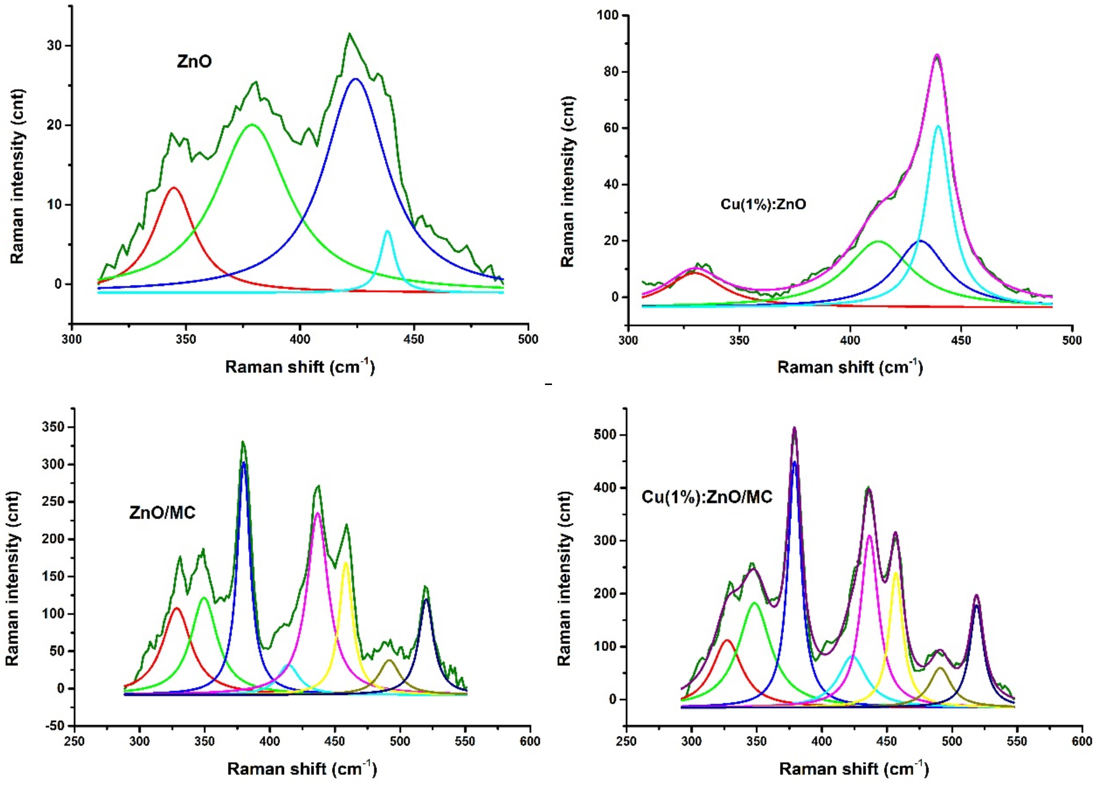

Figure 1. Representative examples of Raman spectra deconvolution for different samples: ZnO, Cu doped ZnO, ZnO/MC and Cu(1%):ZnO/MC

Table S1. S_{BET} (Brunauer-Emmett-Teller) values of the investigated oxide and hybrid NPs

**Sample**	**S_{BET} (m^2/g)**
ZnO NPs	2.8
Cu (1 at.%):ZnO NPs	6.4
ZnO/MC NPs	3.6
Cu (0.1 at.%):ZnO/MC NPs	9.8
Cu (0.5 at.%):ZnO/MC NPs	7.3
Cu (1 at.%):ZnO/MC NPs	4.3

Table S2. ATR-FTIR frequencies characteristic of the MC and hybrid (ZnO/MC, Cu (0.1%):ZnO/MC, Cu(0.5%):ZnO/MC and Cu (1%):ZnO/MC) NPs

**Functional group**	**MC**	**ZnO/MC**	**Cu:ZnO/MC-1**	**Cu:ZnO/MC-2**	**Cu:ZnO/MC-3**
	**Wavenumber (cm^{-1})**				
	**436.20**		**432.90**		
**ZnO**	**559.20**			**509.73**	**509.73**
		829.45	829.53	829.96	829.96
**C-OH**	896.96		885.68	885.23	885.23
		947.12	947.73	946.76	946.76
**C-O-C**	**1028.67**	**1092.05**	**1089.90**	**1089.20**	**1089.20**
	1055.15				
	1157.87				
		1249.31	1249.49	1249.50	1249.50
	**1315.63**	**1349.20**	**1348.77**	**1300.07**	**1300.07**
**C-OH; CH_2**		1453.83	1454.27	1348.85	1348.85
**O-H**		1621.89	1644.17	1454.56	1454.56
		1719.22	1718.60	1645.91	1645.91
	2323.41				
**C-H**	**2886.81**	**2874.30**	**2872.19**	**2872.71**	**2872.71**
**O-H**	**3330.60**	**3415.35**	**3408.70**	**3409.45**	**3409.45**
